# The Role of miRNA in Haematological Malignancy

**DOI:** 10.1155/2013/269107

**Published:** 2013-12-12

**Authors:** Stephanie Gounaris-Shannon, Timothy Chevassut

**Affiliations:** Brighton and Sussex Medical School, University of Sussex, Brighton, East Sussex BN1 9PS, UK

## Abstract

Currently, there are over 1,800 annotated human miRNAs, many of which have tissue-specific expression. Numerous studies have highlighted their role in haematopoietic differentiation and proliferation, acting as master regulators of haematopoietic stem cell function. Aberrant expression of miRNAs has been observed in haematological cancers, exhibiting unique expression signatures in comparison to normal counterparts. Functional and target analyses as well as animal models have attempted to annotate how different miRNA may contribute to the pathophysiology of these malignancies from modulating cancer associated genes, functioning directly as oncogenes or tumour suppressor genes or acting as bystanders or regulators of the epigenetic mechanisms in cancer. miRNAs have also been shown to play a role in modulating drug resistance and determining prognosis between the various subtypes of blood cancers. This review discusses the important role that miRNAs play in haematological malignancies by exploring associations that exist between the two and trying to examine evidence of causality to support the tantalising possibility that miRNAs might serve as therapeutic targets in blood cancers.

## 1. Introduction

The haematological malignancies are a diverse group of neoplastic diseases. They are representing the fifth most common cancer in the UK and are among the twenty most common causes of cancer deaths in the UK [1]. A combination of clinical, cytogenetic, and genetic features and immunophenotype and cell morphology are used to stratify each of the haematological cancers further into subtypes [2]. Tumourigenesis has long been attributed to the alteration of oncogenes and tumour suppressor genes [3]. However, the discovery of non-protein-coding RNA, such as microRNAs (miRNAs) and their possible associations with cancer, has certified suspicions that tumour pathogenicity is far more complex than the simple deregulation of protein-coding genes and warrants further investigation.

**Figure 1:**
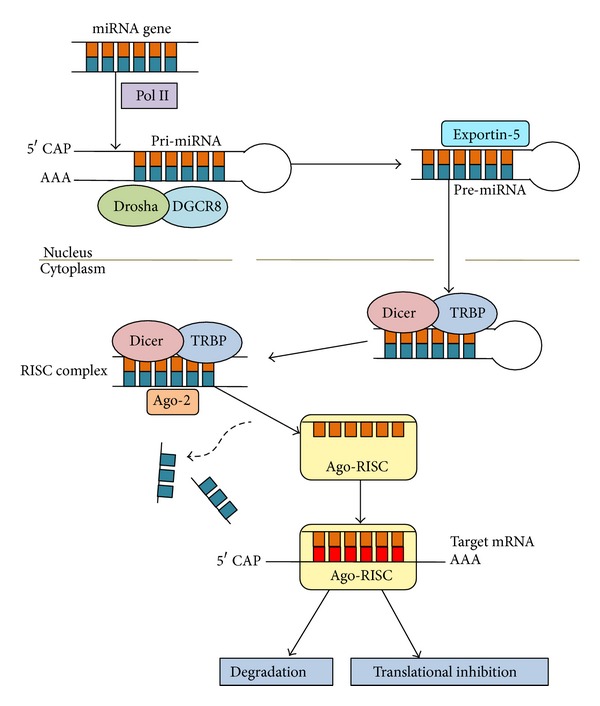
The basics of the biogenesis of miRNA. In most cases, RNA pol II transcribes long primary miRNA (pri-miRNA) transcripts which are cleaved by the RNase III endonuclease Drosha, producing a pre-miRNA. Exportin-5 mediates pre-miRNA exit from the nucleus to the cytoplasm where it is further processed by Dicer, another RNase III endonuclease, producing a 20–22 nucleotide mature miRNA duplex. One strand of the duplex is incorporated into the protein complex RISC (RNA-induced silencing complex) based on its complementarity to the 3′UTR of the mRNA. At this point, the mRNA is either posttranscriptionally silenced or degraded [11].

There are currently 1,862 miRNAs annotated in humans [4], most of which are evolutionarily conserved. miRNAs are 20–22 nucleotide transcripts in their mature form and function to inhibit gene expression via negative regulation of mRNA at a posttranscriptional level [5] (Figure 1). *In vivo* mice studies have illustrated that disruption of miRNA biogenesis through depletion of Dicer leads to embryological and developmental arrest and subsequent incompatibility with life [6]. miRNAs have since been demonstrated to be vital in other essential biological processes such as metabolism [5, 7], proliferation and differentiation [5, 8], apoptosis [5, 9] and haematopoietic lineage differentiation [5]. Considering that proliferation, differentiation, and regulation of apoptosis are fundamental hallmarks of cancer development, it is unsurprising that miRNAs have also been linked to tumourigenesis. The notion that miRNA may be involved in cancer pathogenesis came from studies by Calin et al. who noted that the location of approximately 50% of miRNA genes correlated to cancer-associated genomic regions (CAGRs) or fragile sites [10].

Perturbed miRNA expression in haematological cancers alone cannot certify a direct and causal link between miRNA and cancer development or progression [3]. However, where many studies have solely identified a loose association between certain haematological cancers and aberrant miRNA expression, others have been able to illustrate their role as oncogenic or tumour suppressors in nature through validation of cancer associated targets, and these miRNAs will primarily be the focus of this review.

## 2. miRNA and the Haematological Malignancies

### 2.1. The Chronic Leukaemias

The mass expansion of mature B cells in the bone marrow that characterises chronic lymphocytic leukaemia (CLL) is largely a result of defective apoptosis rather than excess proliferation [3, 12] and many of these cells are halted in cell cycle progression at the G0/G1 phase [12]. Overexpression of the antiapoptotic *BCL2* gene is frequently found in patients [12]. CLL can be broadly divided into two subgroups based on the presence or absence of somatically mutated immunoglobulin variable heavy chain (*IgVH*) genes and levels of zeta-chain-associated protein kinase 70 (ZAP70). These two factors have a widely accepted prognostic value, with patients who exhibit *IgVH* mutations and low ZAP70 levels fairing better [13]. Deletions of 13q14.3, 11q23, and 6q12, as well as trisomy-12, are common abnormalities associated with CLL [12].

**Figure 2:**
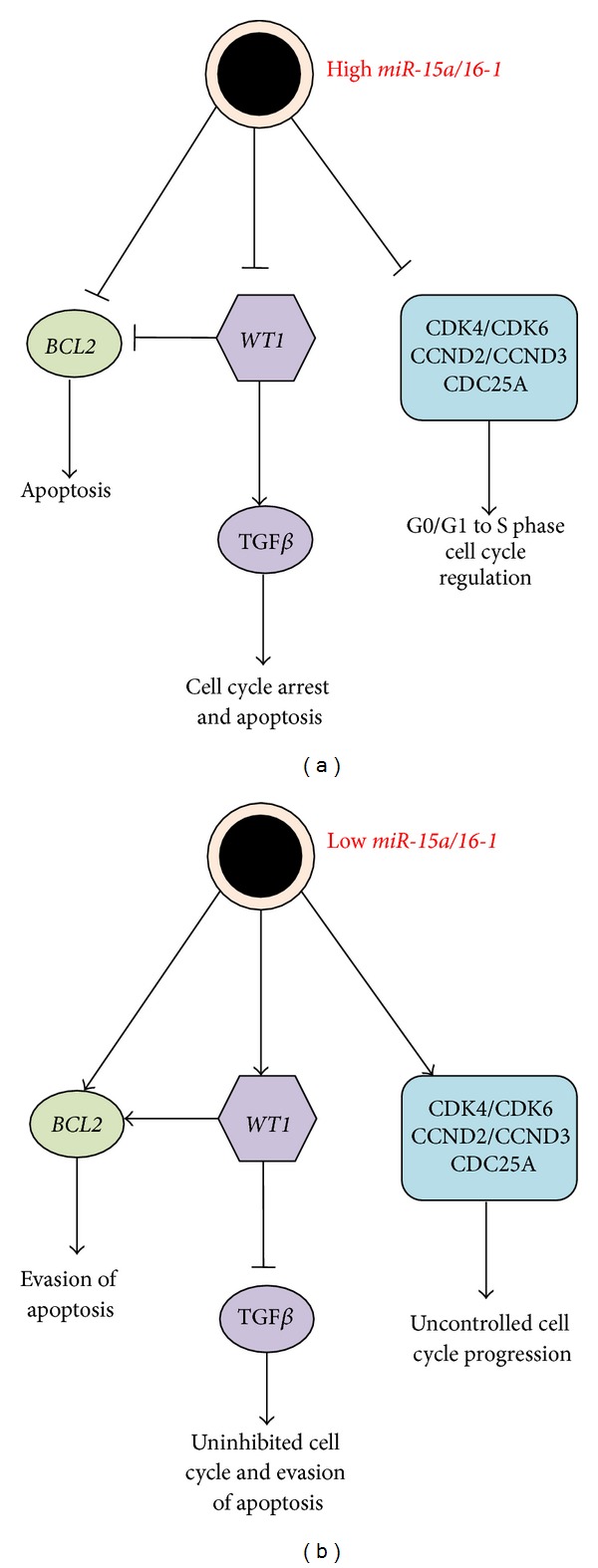
Comparison of possible downstream effects of *miR-15a/16-1* during (a) healthy cells and (b) CLL cells. In healthy cells, high *miR-15a/16-1* expression results in appropriate silencing of targets *BCL2*, *WT1*, and cell cycle factors. In CLL, low *miR-15a/16-1* expression results in uninhibited expression of *BCL2*, *WT1*, and the cell cycle factors resulting in evasion of apoptosis and uncontrolled cell cycle progression.

In an attempt to further understand chromosome 13q14.3 deletions, it was found that *miR-15a/16-1* were the only genes localised to this region [14]. Cimmino et al. demonstrated an inverse correlation between expression levels of *miR-15a/16-1* in CLL cells in comparison to normal CD5+ lymphoid cells extracted from patients or healthy controls, respectively [15]. Complementarity between these miRNAs and *BCL2* was found conserved both in humans and mice [15], and the direct interaction of *miR-15a* and *miR-16-1* was demonstrated through reduced expression of a luciferase reporter construct containing the *BCL2* 3′UTR [15]. To confirm that downregulation of *miR15a/16-1* leads to apoptosis, the same group identified apoptotic DNA fragments in MEG-01 cells transfected with these miRNAs, and subsequent immunoblot assays identified activation of APAF-1-caspase-9-PARP pathway, illustrating that Bcl2 downregulation and subsequent apoptosis by *miR-15a/16-1* are mediated through the intrinsic apoptotic pathway [15]. These data demonstrate that *miR-15a/16-1* may act as bona fide tumour suppressor genes and loss of function of these miRNAs possibly contributes to CLL pathogenesis. Other work has further characterised roles of *miR-15a/16-1* in regulation of cell cycle control, with many predicted targets of these miRNAs acting as important regulators of the G0/G1 transition [16, 17]. Therefore, downregulation of *miR-16* in haematological cancer may contribute to uncontrolled cell cycle progression and tumourigenesis, as well as defective cell apoptosis (Figure 2).

Only 60% of CLL patients demonstrate the 13q14.3 deletion [18], meaning that high *BCL2* expression in most patients cannot be explained by *miR15a/16-1* downregulation alone. Additionally, the majority of 13q14.3 deletions are monoallelic [16], suggesting additional aberrations of the remaining alleles are required in order for *miR-15a/16-1* to fulfil roles as tumour suppressor genes according to Knudson's two-hit hypothesis [19]. An explanation might lie in the presence of miRNA-associated mutations. In the CLL-prone New Zealand black (NZB) mouse model, reduced expression of *miR-16-1* was identified secondary to a point mutation in the 3′DNA adjacent to the *miR-16-1* region [20], and Calin et al. demonstrated that a minority of CLL patients possess germline mutations in *pre-miR-15a/16-1* that affect subsequent processing [21]. Despite controversy over the mechanisms by which *miR-15a/16-1* mediate their effects, it is clear that these miRNAs play an important role in CLL pathogenesis.

T-cell leukaemia-1 gene (*TCL1*) drives tumourigenesis by activating the oncogene *AKT*, driving proliferation and cell survival [22]. It plays a pathological role in CLL [23], found highly expressed in patients with 11q deletion [24] and correlating with aggressive malignancy (high ZAP70 levels and unmutated *IgVH*) [23, 24]. Overexpression of *TCL1* in these cases might be explained by a downregulation of *miR-181a* and *miR-29b*, since both act to target *TCL-1* [23]. *miR-29b* also targets antiapoptotic myeloid cell leukaemia sequence 1 (*MCL1*) [25], an important lymphocytic survival factor associated with CLL [26]. These tumour suppressor miRNAs may make optimal treatment options for *TCL1* or *MCL1* expressing CLL patients [12]. Interestingly, *miR-181a* has conversely shown upregulation in different morphological subtypes of acute myeloid leukaemia (AML) [27], demonstrating that certain miRNAs may have dual functions (tumour suppressor or oncogenic) depending on cell type and context.

Abnormalities of the tumour suppressor gene tumour protein 53 (*TP53*) can be found in CLL patients with chromosome 17p13 aberrations [13]. Using real-time quantitative polymerase chain reaction (qRT-PCR), patients with *TP53* abnormalities were analysed for the expression of 35 miRNAs. Downregulation of p53-regulated *miR-34a* was demonstrated [13]. Several genes involved in cell cycle progression and linked to tumourigenesis (*CDK4*, *CDK6*, *MYCN*, *Cyclin-E*, and *E2F1*) have been validated as direct targets of *miR-34a* [13]. Additionally, levels of *miR-29c* and *miR-17-5p* were also reduced. The latter is part of the *miR-17-92* cluster and has been shown to target *E2F1*, *p21*, and *Cyclin-D1* [28], suggesting a role as a tumour suppressor. A different study conversely showed upregulation of *miR-34a* and members of the *miR-29* family [29], and high expression of *miR-331* in CLL patients, suspected but not validated to target suppressor of cytokine signalling 1 (*SOCS1*) [29, 30]. Reduced SOCS1 levels have been previously found in cancers [31] and this would prevent negative feedback of the JAK/STAT pathway and might allow cell proliferation, cell survival, and angiogenesis to progress uninhibited [32].

Analysis of miRNA in CD19+ cells from peripheral blood of CLL patients and healthy controls revealed deregulation of various miRNAs, including *miR-155*, *miR-150*, *miR-21*, and *miR-101* [33]. Although potential targets were not discussed within this study, some have previously shown experimentally validated targets.  Table 1 defines directly downregulated targets validated by luciferase reporter assays as determined by TarBase [30]. Particularly, the downregulation of PU.1 by *miR-155* may be of relevance in CLL as multiple studies have identified a correlation between PU.1 and lymphoid tumourigenesis [34].

Chronic myeloid leukaemia (CML) is a clonal malignancy of predominantly myeloid cells characterised by a chronic phase (CML-CP) followed by an accelerated phase and blast crisis (CML-BC) [35]. A hallmark of CML is the well-characterised t(9; 22) aberration, otherwise known as the Philadelphia chromosome [35], which results in the *BCR-ABL* fusion gene in over 95% of patients [36]. This fusion protein is susceptible to tyrosine kinase inhibition, and subsequently imatinib mesylate has been the gold standard treatment [37]. However, evidence shows imatinib is unable to fully inactivate *BCR-ABL* signals [36] and a number of patients are resistant to this therapy [37], highlighting the need to further understand the biology of CML.

Using qRT-PCR, Agirre and colleagues analysed 157 miRNAs in CD34+ cells between CML patients and healthy donors. They found one upregulated miRNA (*miR-96*) and four downregulated (*miR-151*, *miR-150*, *miR-125a*, and *miR-10a*) that were able to consistently differentiate CML cells from normal CD34+ cells in two separate cohorts [36]. Treatment of the Mo7e leukaemic cell line with imatinib correlated with an upregulation of *miR-150* and *miR-151*, suggesting that in CML these miRNAs might be downregulated as a consequence of BCR-ABL1 activity [36]. The same investigations suggest *miR-10a* is downregulated in CML patients independent of BCR-ABL activity [36]. This might help to explain the persistence of disease in some patients despite imatinib therapy. In line with this hypothesis, reexpression of *miR-10a* in CML CD34+ cells inhibited cell growth, demonstrating its potential role as a tumour suppressor in CML [36]. Gene array studies have identified high expression of the transcription factor upstream stimulatory factor 2 (USF2) in CML patients, and *USF2* has been proposed as a *miR-10a* target through computational algorithms [36]. An association between the two was demonstrated, as colon carcinoma RKO cells transfected with *pre-miR-10a* showed a significant decrease in mRNA levels of *USF2* [36]. Furthermore, predicted base pairing between USF2 and miR-10a showed perfect complementarity and luciferase reporter assays revealed that renilla luciferase activity of the USF2 3'UTR vector was significantly reduced in RKO cells when transfected with miR-10a [36].

The *BCR-ABL* fusion gene also upregulates the *miR-17-92* cluster [38]. This cluster is conserved in the chromosome 13 open reading frame 25 (c13orf25) genomic region of chromosome 13q31-32 and is transcriptionally regulated by c-myc, which works cooperatively with *BCR-ABL* in CML pathogenesis [38]. The cluster likely has an oncogenic role in CML as overexpression induced proliferation *in vitro* [38]. Interestingly, *miR-17-92* showed high expression in CML-CP, but not in blast crisis CML-BC [38], despite this phase being characterised by increased *BCR-ABL* activity [39]. Different members of the *miR-17-92* cluster affect genes whose inhibition results in proliferation and inhibition of apoptosis, such as E2F1, PTEN, and BIM [40]. These experiments give an initial insight into different pathological mechanisms between CML-CP and CML-BC.

A novel role of miRNA has been identified during blast crisis. In this phase, increased *BCR-ABL* activity results in aberrant activity of RNA binding proteins (RBPs) [39]. One RBP affected is hnRNPE2 which arrests myeloid differentiation through interaction with C/EBPa [41, 42]. Micro-array, northern blot, and qRT-PCR approach identified *miR-328* as downregulated in CML-BC secondary to BCR-ABL activity both *in vitro* and *in vivo* [39]. Postulating that miRNA and RBPs may interact, Eiring et al. showed that loss of *miR-328* in CML-BC is regulated by hnRNP-E2, as an inverse correlation between the two exist, suggesting hnRNP-E2 may mediate *miR-328* activity. They identified that the two can interact and do so in a seed sequence independent manner. Reexpression of *miR-328* restores differentiation and impairs blast survival not only through traditional seed sequence dependent posttranscriptional silencing of the prosurvival factor PIM1, but also through interacting with hnRNP-E2 (PCBP2), acting as a decoy to rescue C/EBPa mRNA translation and function [39]. Another RBP, hnRNP-A1, is also upregulated in CML-BC and has been shown to bind *pri-miR-17-92* [43], which might attempt to explain a lack of *miR-17-92* expression in CML-BC, as described previously.

### 2.2. The Acute Leukaemias

The chromosomal aberrations in acute myeloid leukaemia (AML) are diverse. Some of the common abnormalities include trisomy-8, monosomy-7, translocations such as t(15; 17), t(8; 21), and the inversion of chromosome 16 [35, 44, 45]. The latter three alterations generate the fusion proteins *AML-ETO*, *PML-RARa,* and *CBFb-MYH11*, respectively [31]. Many patients with AML have a normal karyotype and less than one-half of these patients suffer with internal tandem duplication mutations of FLT3 (FLT3-ITD+), a membrane associated tyrosine kinase, which is associated with poor overall survival [31, 35]. A smaller proportion exhibit mutations of C/EBPa, and the majority demonstrate alterations in the nucleophosmin (*NPM1*) gene. In comparison to FLT3-ITD+ patients, the presence of *NPM1* mutations alone offers a better prognosis [35].

Much like AML, acute lymphocytic leukaemia (ALL) is a heterogeneous clonal neoplastic disease of the bone marrow, lymph nodes, thymus, and spleen [46]. It can be subdivided into T-cell or B-cell ALL, both of which are further defined by morphology, immunophenotype, and clinical features as well as cytogenetic aberrations [46]. Over 7,000 cytogenetically abnormal childhood and adult cases of ALL have been identified, most of which involve the B-cell lineage [47]. The prevalence of these chromosomal aberrations differs vastly between adult and child ALL, as is the case with AML [46]. For example, t(4; 11) which disrupts the mixed lineage leukaemia (*MLL*) gene is fourfold more common in children than adults and t(15; 17), the most common chromosomal alteration in AML, is never seen in infants under 12 months with AML [48]. ALL is the most common type of childhood cancer, but despite widespread efforts into treatment approaches, up to one-third of patients relapse and many causes of the malignancy are still undetermined [49]. Evidence suggests that different chromosomal and genetic subtypes of AML and ALL are associated with differential miRNA expression. Analysing the role of miRNA in different leukaemic subtypes may further our understanding of the oncogenic process in the AML and ALL [49].

A number of deregulated miRNAs in AML have been shown to be involved in haematopoiesis (*miR-130a*, *miR-10a*, *miR-181*, *miR-451*, and *miR-155*), suggesting that their aberration might be involved in the oncogenic process [50]. Low levels of *let-7b* and *-c* have been identified in different subtypes of AML [51], and the causal relationship of its deregulated expression in cancer can be supported by evidence suggesting it targets a number of genes involved in the cancer process such as *KRAS*, *TRIM71*, *nRAS*, *MYC*, and *HMGA2* [31].

*miR-10a*, *miR-10b*, and members of the *miR-29* family were shown to be overexpressed in mutated *NPM1* AML patients [52]. The genes of *miR-10a* and *-b* are located within the *HOX* clusters and upregulation of these two miRNAs has been seen in cytogenetically normal AML (CN-AML) patients in association with *HOX* gene upregulation [53], suggesting that miRNA may be regulated by expression of neighbouring genes. In a study of 30 AML patients, *HOXA1*, *HOXA13*, *HOXB1*, and *HOXB13* were the only *HOX* genes not to show strongly positive or negative correlation with *miR-10a* and *miR-10b* [54]. In contrast, these miRNAs have shown to directly target *HOXA1* in the K562 cell line [55]. This might be explained by the small sample size of the patient study or the fact that the correlation between miRNA and *HOX* genes is specific to species and tissue type [53]. Although the role of *HOX* genes in cancer biology has not been fully defined [53], evidence suggests HOX genes may prevent apoptosis [55] and *HOXA9* has been associated with poor prognosis in AML [56].

Illustrating that differential miRNA expression might be dependent on AML subtypes, *miR-29* has conversely shown downregulation in *MLL*-rearranged AML patients [31]. It has been suggested that *miR-29* may play a role in tumourigenesis by inhibiting apoptotic genes such as *MCL1*, and luciferase vector assays have validated the direct binding of *miR-29b* to the 3′UTR of *MCL1* [57]. In line with this theory, cells from an AML patient cohort underwent apoptosis upon reexpression of *miR-29b*. Upregulation of *miR-24* in AML patients with t(8; 21) is thought to be caused by binding of the *AML-ETO* fusion product to the miRNA gene locus, and overexpression leads to the downregulation of mitogen-activated protein kinase phosphatase 7 (MPK7) [58]. Subsequent lack of inhibition of both c-Jun is thought to cause uncontrolled cell growth in myeloid leukaemia cells [58].

Also implicated in AML is the *miR-15a/16-1* cluster, shown to act as tumour suppressor genes [59]. This effect might be mediated through the targeted silencing of *BCL2*, programmed cell death protein 4 (*PDCD4*), and Wilms tumour 1 (*WT1*) [59, 60], the latter of which is frequently detected at high levels in AML [60]. An inverse correlation between this miRNA cluster and WT1 levels was shown in primary AML blasts in comparison to their normal counterparts [59]. However, luciferase reporter assays identified that this association is not mediated directly through miRNA translational silencing of the *WT1* mRNA [59] and further research into the mechanisms of *WT1* silencing in AML patients is warranted.

The *miR-17-92* has been reported to act as an oncogene in lymphoid tissue [61], and *miR-17-19b* is associated with the downregulation of p21, an inhibitor of cell cycle progression [62]. In one study, murine myeloid progenitors were cotransduced with the *MLL*-rearrangement (*MLL-AF10*) and *miR-17-19b*, and then transplanted into lethally irradiated mice. The leukaemia that subsequently developed was more aggressive than that in mice without *miR-17-19b* expression [62]. Along with 19 other genes, *p21* is a predicted target of *miR-17-92* and *p21* 3′UTR shows perfect complementarity to the seed region of *miR-17-5p* [62]. Knockdown of *p21* in *MLL*-transduced murine myeloid progenitors mimicked the effect of *miR-17-19b* overexpression in *MLL*-rearranged leukaemia cells [62]. In T-ALL without *MLL*-rearrangements, *miR-17-92* has also shown oncogenic potential as induced expression of *miR-19* accelerated the development of ALL in mice [63]. As with many targets of miRNA, four confirmed targets of *miR-19* (*Bim*, *PTEN*, *PRKAA1*, and *PP2A*) act to negatively regulate the phosphatidylinositol-3-kinase (PI3K) pathway [31], suggesting that the oncogenic effect of overexpressed *miR-17-92* in acute leukaemias may be related to the silencing of these genes.

### 2.3. Lymphomas

**Figure 3:**
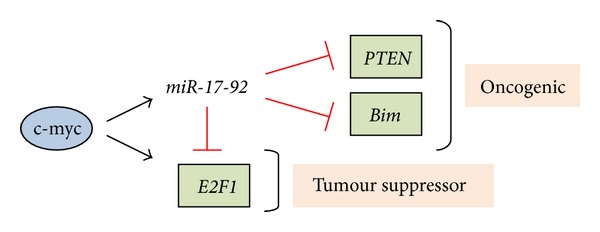
The dual oncogenic and tumour suppressor nature of *miR-17-92*. The transcription factor c-myb induces expression of both *miR-17-92* and *E2F1*. *MiR-17-92* can subsequently act both as a tumour suppressor gene through inhibition of *E2F1* or as an oncogene via the targeted silencing of tumour suppressor genes such as *PTEN* and *Bim*.

In B-cell lymphomas the c13orf25 gene located in chromosome 13q31-32 is frequently amplified [64]. Located within this region is *miR-17-92*, and its subsequent overexpression has been shown to play an oncogenic role in these malignancies. Overexpression of *miR-17-92* in B-cell lymphoma *MYC* TG mice accelerated formation of lymphoid malignancies and mice death [65]. Previously discussed targets of this cluster include the tumour suppressor *PTEN*, the proapoptotic *Bim*, and the cell cycle regulator *E2F1*. Immunoblot analysis and luciferase reporter assays have confirmed *PTEN* and *Bim* as direct targets of cluster members *miR-17-5p/-19* and *miR-92*, respectively [66], supporting an oncogenic role for this cluster. In contrast, targeting *E2F1* gene suggests a tumour suppressor role. This gene, like *miR-17-92*, can be directly upregulated by c-myc. Interestingly, expression of *miR-17-92* in HeLa cells demonstrated a 50% decrease in E2F1 protein levels, and *E2F1* has experimentally been validated as a direct target of the cluster members *miR-17-5p* and *-20a* [67]. This illustrates a mechanism in which *miR-17-92* can tightly regulate c-myc oncogenesis and also acts as a tumour suppressor gene (Figure 3). Therefore, like many other miRNAs, *miR-17-92* possesses both oncogenic and tumour suppressor functions depending on the specific biological context [64]. Interestingly, E2F1 can act to increase proliferation in some settings and induce apoptosis in others [68, 69]. If in B-cell lymphomas it acts to induce apoptosis, its downregulation by *miR-17-92* might further define this cluster as an oncogene.

The overexpression of *miR-155* in lymphomas followed identification of the B-cell integration cluster (*BIC*) gene as a common retroviral integration site in avian leucosis virus (ALV) induced B-cell lymphomas [64], where *BIC* represented a conserved stretch of nucleotides corresponding to *pri-miR-155* [70]. Since a 10–60 fold-increase in miR-155 expression has been identified in aggressive DLBCL [71] and expression of this miRNA in NK-cell lymphomas has been associated with decreased levels of SHIP, PTEN, and PDCD4, all of which are tumour suppressors, suggesting that inhibition of *miR-155* in this T-cell lymphoma drives tumourigenesis [72]. It is unsurprising that *miR-155* plays a role in B-cell malignancy considering overexpression of this miRNA in TG mice led to a preleukaemic B-cell proliferation [73]. This might suggest *miR-155* plays a role early in oncogenesis before secondary events lead to occult malignancy. The regulation of PU.1 by *miR-155* may be of important pathogenic significance as this transcription factor regulates late-onset differentiation of B cells [74].

Interestingly, much like differences in miRNA expression have been identified between adult and childhood acute leukaemias, one study identified a 100-fold upregulation of *miR-155* in paediatric Burkitt's Lymphoma but not in adult cases [75]. *miR-155* deregulation has also shown upregulation in all other haematological malignancies described in this review suggesting that this miRNA may have multiple oncogenic functions in different cell types or that all malignancies might share a common target fundamental to tumour suppression [75].

### 2.4. Multiple Myeloma

Multiple myeloma develops secondary to a benign monoclonal gammopathy of undetermined significance (MGUS) [76]. Full-blown malignancy is characterised by excessive proliferation of plasma cells that infiltrate the bone marrow and produce abnormal immunoglobulin (M protein) [77].

The survival and expansion of malignant plasma cells are dependent on signal transducer and activator of transcription 3 (STAT3), secondary to IL-6 signalling [77]. It has been found that upregulation of the gene encoding the oncogenic *miR-21* is also STAT3 dependent and overexpression can promote survival of myeloma cells in the absence of IL-6 [78]. The contribution of *miR-21* to STAT3 oncogenic activity in myeloma is supported by frequent upregulation of this miRNA in both MGUS and myeloma patients in comparison to healthy donors [76, 77]. SOCS proteins mediate inhibition of the STAT pathways, and downregulation of SOCS1 is frequent in multiple myeloma [76]. High expression of both *miR-19a* and *miR-19b*, members of the *miR-17-92* cluster, also targets SOCS1 in myeloma cells [76].

Pichiorri et al. showed upregulation of the *miR-106-25* cluster in MGUS patients [76]. Although the sample size was limited in this study (*n* = 6), the findings illustrate deregulation of oncogenic miRNA early in disease progression [76]. The oncogenic potential of *miR-106-25* and *miR-21* may be due to targeted downregulation of tumour suppressors such as *PTEN*, *Bim*, and *p21* [77]. Through targeting of the latter two, *miR-106-25* has also shown to suppress transforming growth factor beta (TGFb), which is responsible for cell cycle arrest and apoptosis [79]. Interestingly, *miR-106-25*, along with *miR-32* and *miR-181a/b,* may target the p53 regulator p300-CBP-associated factor (*PCAF*), which would reduce tumour suppressor p53 levels in multiple myeloma [77].

*miR-15a/16-1* are also deregulated in over 50% of myeloma patients [77], and patient studies showed significant downregulation of *miR-15a/16-1* in CD135+ myeloma cells in comparison to normal counterparts [80, 81]. Interestingly, deletion of chromosome 13q14.3 which encodes these two miRNAs is not significantly associated with myeloma patients over normal controls [77], and therefore the genetic basis of their deregulation in myeloma is yet to be fully determined. At the molecular level, *miR-15a/16-1* have been shown to inhibit *cyclin-D1*, *cyclin-D2*, and *CDC25A*, key regulators of cell cycle progression [77], as well as protein kinase B (*AKT*) and nuclear factor-kappa B (NF-*κ*B) pathways involved in oncogenesis [80]. Most cases of multiple myeloma demonstrate deregulation of one or more *cyclin-D* genes [82], which in this case might be explained by reduced *miR-15a/16-1* levels. Combined with the role of these miRNAs in lymphomas and leukaemia, *miR-15a/16-1* provide evidence that a single miRNA can have multiple and complex effects in cell biology [77].

## 3. Interpretation and Analysis

This review highlights that certain miRNAs may act as bona fide tumour suppressor genes or oncogenes, alongside evidence that supports certain miRNAs to have the potential to act depending on both cell type and context (Table 2).

It has been suggested that four types of evidence can strengthen the argument for miRNA acting directly as tumour suppressors or promoters (Table 3) [75]; not only was the identification of *miR-15a/16-1* the first reported link between miRNA and cancer [12], but also continuous research has allowed these miRNAs to fulfil all four of these criteria. They have been found deregulated in a diverse range of cancers, including CLL, myeloma, lymphomas, and prostate cancer [1, 83, 84], absent or reduced expression has been found secondary to deletion of the *DLEU2* gene at chromosome 13q14.3 [19] and/or mutational change [2, 20, 21], evidence for their natural tumour suppressor function has been supported through various xenograft mouse models and in the CLL-prone NZB mouse model [3, 20, 21], and the antiapoptotic protein Bcl2 [15], as well as others [60, 77], has been experimentally validated as a direct target of *miR-15a/16-1* [4]. Although many miRNAs analysed in this review may fulfil one or two of these criteria, *miR-17-92* and *miR-155* are other miRNAs involved in haematological malignancy able to fulfil all four of these criteria.

Understanding how miRNAs contribute to the six hallmarks of cancer may also provide solid evidence for their role in tumourigenesis. This review makes it clear that miRNAs greatly contribute to enhanced proliferation, differentiation, and reduced apoptosis in haematological malignancies. Angiogenesis has been highlighted as an important feature of haematological malignancies, providing a specialised environment for HSC proliferation and differentiation through provision of GM-CSF, G-CSF, and IL-6 shown to be important in the proliferation and survival of malignant cells [85]. The oncogenic *miR-17-92* cluster and the tumour suppressors *miR-15a/16-1* have been shown to be proangiogenic and antiangiogenic, respectively [86, 87]. If miRNAs are able to promote bone marrow vascularisation in this way, the secretion of GM-CSF, G-CSF, and IL-6 from these areas of high vascular density might support a role of miRNA in self-sufficiency in growth signals, another hallmark of cancer. Metastasis of haematological cancers to other organs is a rare phenomenon [88]; these data highlight a role for miRNA in most hallmarks and increase support for a fundamental role in oncogenesis.

Additional support for the role of miRNAs in cancer comes from their association with epigenetic changes. DNA CpG island hypermethylation of tumour suppressor genes is a well-characterised aberration in malignant cells, along with global genomic hypomethylation and histone modification disturbances [89]. Over half of miRNA genes are associated with CpG islands [90]. If these miRNAs are bona fide tumour suppressor genes, their hypermethylation and subsequent transcriptional silencing will affect cancer progression [91]. Downregulation of *miR-34* due to promoter hypermethylation has been identified in primary samples of CLL, myeloma, and NHL [92]. The *MIR-34a* gene is located at chromosome 1p35, which is subject to loss of heterozygosity in various cancers [92]. Loss of heterozygosity together with promoter hypermethylation of this gene fulfils Knudson's two-hit hypothesis [19] for bona fide tumour suppressor genes.

Paradoxically, miRNA may also directly target epigenetic machinery [31]. Evidence suggests that *miR-29b* may target the DNA methylation agents DNMT1 and DNMT3 [93]. Reexpression of *miR-29b* in AML cells resulted in global DNA hypomethylation and subsequent upregulation of tumour suppressor genes [93]. Further evidence for the association between miRNA and cancer epigenetics is vast and beyond the scope of this review.

Importantly, miRNAs are increasingly recognised as prognostic biomarkers and therapeutic targets in various types of haematological malignancies. Briefly focusing on AML, a few recent studies have attempted to support this notion. It was found that *mir-155* upregulated in patients with CN-AML was associated with lower remission rates and higher overall survival rates [94]. The high expression of *miR-155* was interestingly associated with genes widely known to be deregulated in AML supporting the role of this miRNA in AML pathogenesis [94]. Recent work on the role of *let-7a* showed enhanced chemosensitivity of AML cell lines overexpressed with *let-7a* [95]. A miRNA microarray study using 15 chemoresistant and 18 chemosensitive patients with AML identified three consistently upregulated miRNAs associated with therapeutic response. One of these, *miR-363*, was further characterised to target the genes RGS17 and HIPK3, which have previously been associated with therapeutic response in AML patients [96]. These studies identify that certain miRNA signatures are associated with the clinical outcome.

There are caveats and difficulties faced within the field of miRNA research. The benefits of investigating primary cells and patient cohorts are often reduced due to small sample size, reducing external validity. As mentioned in AML and ALL, differences in cytogenetics and therefore miRNA expression may be age dependent within the same malignancy. Often studies broadly look at disease without separating findings to a particular cytogenetic subtype or age group. A study looking at differences of miRNA expression between adult and childhood acute leukaemias [31] shows that this is an important consideration to take into account when analysing the function of specific miRNAs. Many studies base much of their interpretation on array- and PCR-based miRNA profiling studies, which have the disadvantage of having to already predict which miRNAs are going to be deregulated. This makes finding novel miRNA associated with haematological malignancy difficult [97]. Often authors search for miRNAs that have shown deregulation in solid tumours, a method that can miss miRNAs important to haematological cancers. This is of particular relevance considering many miRNAs have shown to be tissue-specific and dependent on cellular context, as previously discussed in this review [97]. It is also important to note that not all studies reach agreement about miRNA expression in various haematological malignancies. Differences in methodology might explain this, as many studies likely use different microarray platforms. A study into the sources of data variability across microarray platforms identified poor reproducibility between platforms and across laboratories [98]. Different results in miRNA expression might also be secondary to differences in patient samples, as well as the fact that not all studies used the same cell lines when examining miRNA *in vitro*. Additionally, in some studies cell lines were used that were not indicative of the primary disease. The colonic cancer RKO cell line was used to investigate *miR-10a* in CML by Aggire et al. [36], and many functional studies of *miR-15a/16-1* and *BCL2* used nonlymphoid cell lines [16]. Different groups may have used cells at different maturation stages, which may have an effect on miRNA expression since *miR-155* and *miR-106-25* have shown effect early in disease progression of lymphomas and myeloma, respectively [73, 76]. These problems call for the need of standardised methodology for miRNA investigation and an evaluation of the appropriate experimental approaches to study miRNA in haematological cancers.

## 4. Concluding Remarks

It is apparent that there is accumulating evidence for the role of miRNA in haematological malignancies, with different patterns of expression correlating with distinct types and subtypes. In this review, evidence from *miR-181* and *miR-17-92* illustrates how miRNA can function both as tumour suppressors and promoters dependent on biological and cellular context. The well-characterised functions of *miR-15a/16-1* demonstrate that a single miRNA can have far reaching and multiple effects in cells and in cancer progression, targeting a variety of cancer associated genes. Furthermore, expression analysis has revealed that certain miRNAs, such as *miR-155*, are deregulated in all haematological malignancies, suggesting that certain miRNAs may be fundamental to tumourigenesis.

Our understanding of these small noncoding RNA is continually evolving and more recent research highlights areas of miRNA regulation and function not previously considered or identified. miRNAs were initially discovered as posttranscriptional silencers of protein-coding genes, and this still remains true. However, their function and mechanisms by which they mediate their effects extend further than this. As well as acting through the RISC complex, they have been shown to mediate protein inhibition through epigenetic modifications of proteins involved in tumourigenesis or tumour inhibition [97]. Evidence also suggests miRNA may function as a decoy for other noncoding proteins, as discussed for *miR-328* in CML-BC [39]. Additionally, other groups have identified seed sequence mutations in miRNA, which may act to alter target specificity [99]. Finally, Jing et al. demonstrated an extended role of *miR-15a/16-1* showing that in addition to posttranscriptional silencing, *miR-16* may function to target AU-rich elements (AREs) in target mRNA, mediating their decay [100]. Of huge interest is the identification of other highly conserved noncoding RNA, such as ultraconserved regions (UCRs) [101], which have shown functional importance in cell biology [102] and have shown cancer-specific signatures, including within haematological malignancies [103]. This provides an additional layer of insight into the nature of tumourigenesis, especially considering that miRNAs and UCRs have been shown to interact [104], which may influence complex pathways associated with cancer formation and progression. Current data and our overall understanding of miRNA regulation and function have likely only been touched upon. What is clear is that miRNAs reflect an additional layer of insight into the haematological malignancies, and future investigations into their role are warranted given their promise for therapeutic opportunities.

## Figures and Tables

**Table 1:** Experimentally validated targets of miRNA overexpressed in Fulci et al. [33].

	Experimentally validated targets (derived from TarBase [30])
*miR-150 *	MYB and c-myb

*miR-155 *	AGTR1, SMAD2, TP53INP1, BACH1, TAB2, SHIP1, Ets-1, JARID2, DHX40, NARS, ARID2, TRIP13, PHC2, PKN2, PKIalpha, NF-*κ*B, PICALM, KBTBD2, UBQLN1, ETS1, PALD1, DET1, HIVEP2, LRRC59, ARL15, CSNK1A1, TLE4, MRPS27, ZNF652, C16orf62, ZKSCAN5, FAM177A1, PDCD4, MASTL, ZNF273, MED13L, C3orf18, GATM, E2F2, NDS3, VPS18, MCM8, ZNF28, PHF14, PAK2, SMARCA4, SBP2, SAC2, HBP1, LKAP, CCDC41, KLHL5, WEE1, DCUN1D2, AGO4, WWC1, FAM91A1, MPP5, MRPL18, TM6SF1, APAF1, ARMC2, LCORL, RAB11FIP2, DRE2, WBP1L, RAPGEF2, TRAK1, ZNF254, CEP41, LDOC1, TBC1D14, CYP2U1, IL17RB, MAP3K10, INTS6, FAM199X, INTS6, TSPAN14, PRKAR1A, SAP30L, TAF5L, MATR3, LRIF1, MBNL3, MORC3, PHF17, CLUAP1, CARD11, LNX2, LSM14A, PCDH9, EXOSC2, MEF2A, CARHSP1, ANAPC16, NFAT2CIP, PU.1, c-myb, C/EBP, BC0RL1, SHIP, RREB1, GNAS, NIK, and GPM6B

*miR-21 *	PDCD4, PTEN, RECK, PPARa, TIMP3, TPM1, FasL, TGFBR2, SERPINB5, Pdcd-4, and CDK2AP1

*miR-101 *	MYCN, ATP5B, EZH2, and MCL1

**Table 2:** Predicted role in oncogenesis of miRNAs analysed within this review.

Tumour suppressor	Oncogenic	Dual role in different haematological subtypes
*miR-15a/16-1 *	*miR-155 *	*miR-17-92 *
*miR-29b *	*miR-96 *	*miR-29c *
*miR-34a *	*let-7 *	*miR-181a/b *
*miR-151 *	*miR-24 *	*miR-150 *
*miR-204 *	*miR-21 *	*miR-125 *
	*miR-32 *	*miR-10a *
	*miR-106-25 *	*miR-196 *

**Table 3:** Evidence to strengthen association of miRNA with tumourigenesis [75].

1	Widespread deregulation in multiple cancers

2	Gain or loss of miRNA function secondary to deletions, mutation, or amplifications

3	Documentation of tumour suppressor or promoter activity in animal models

4	Verification of cancer-relevant targets in order to identify likely mechanisms through which miRNAs promote oncogenesis
